# The role of endocannabinoid signaling in the cytoskeleton functionality in migrating neurons

**DOI:** 10.18103/mra.v13i10.7041

**Published:** 2025-10

**Authors:** Yury M. Morozov, Pasko Rakic

**Affiliations:** 1 Department of Neuroscience, Yale University School of Medicine and Kavli Institute for Neuroscience, New Haven, Connecticut 06510, USA

**Keywords:** cannabinoid type 1 receptor, nuclear envelope rupture, cell membrane rupture and repair, accidental cell death, brain development pathology, recovery of neurons

## Abstract

A fraction of neurons migrating through the developing brain are known to show nuclear envelope rupture and herniation of the chromatin in cytoplasm. We recently reported powerful streams of chromatin rupturing nuclear envelopes together with the plasma membranes in migrating cerebral neurons in mouse embryos. Such chromatin streams represent a novel form of cell pathology, which we named ‘piercing nuclear hernia’ (PNH). Simultaneous piercing of the nuclear and plasma membranes exposes nucleoplasm and cytoplasm to the intercellular space and may result in accidental cell death which, in contrast to the programed cell death mechanisms, are not detectable using biochemical or immunochemical markers for apoptosis, autophagy, or necrotic type of cell death. We also showed that the disfunction of the endocannabinoid system increases the probability of nuclear membrane rupture and chromatin herniation in developing brain. Indeed, about 40% of migrating neurons in cannabinoid type 1 receptor knock-out mouse embryos and wild type embryos exposed to two different agonists of the cannabinoid receptor show nuclear envelope ruptures or/and PNHs. This indicates that deviations from optimal functioning of the endocannabinoid system in under- or over-activity may trigger analogous mechanisms increasing the membrane’s vulnerability and chromatin herniation. The role of increased intranuclear pressure and cytoskeleton malfunction in the mechanism of nuclear envelope rupture is documented and commonly accepted. In accordance, our results provide evidence that optimal endocannabinoid signaling plays a role in cytoskeleton functionality in migrating neurons. In a fraction of neurons, catastrophic rupture of the nuclear and plasma membranes provokes ultrastructural pathology in the mitochondria and other organelles. At the same time, other neurons with PNH show generally normal ultrastructure that may indicate a mechanism of neuronal cell body repair. Further studies of neuronal cell body recovery may identify yet unknown molecular mechanisms and become instrumental for increasing regenerative capacity of neurons during neurodegenerative diseases, after traumatic brain injury and ischemic conditions. On the other hand, the demonstrated novel pathology of PNH in migrating cells and the procedure of its upregulation may be useful for inducing breaks of the plasma membrane and death of metastatic tumor cells.

## Introduction

Medical cannabis products are approved or tolerated in many countries and, increasingly, self-administered by pregnant individuals seeking relief from pain, nausea, or depression. The use of marijuana and synthetic cannabinoids during pregnancy and lactation poses potential risks for intrauterine and postnatal brain development, particularly as cannabinoids can appear in breast milk. The extent to which marijuana and medicinal cannabinoids affect immature brains is still the subject of debate and extensive biomedical studies [Reviewed in^1–4^].

The endocannabinoid system is involved in several cognitive and physiological processes, including cardiovascular regulation, fertility, pregnancy, prenatal and postnatal development, activity of the immune system, food consumption and energy metabolism^1,5–12^. Endocannabinoid signaling via cannabinoid type 1 receptor (CB_1_R) participates in retrograde synaptic modulation but also involves non-synaptic pathways^13–15^. In addition to abundant expression in the axonal plasma membrane, CB_1_R accumulates in the membranes of intracellular vesicles in the cell bodies of adult animals. As demonstrated in rodent and rhesus macaque embryos, CB_1_R also accumulates in immature interneurons tangentially migrating through the embryonic cerebral marginal zone and projection neurons vertically migrating through the intermediate zone and cortical plate^16–20^. One demonstrated role of CB_1_R, which putatively involve the intracellular vesicles, is self-inhibition of cholecystokinin-expressing interneurons and a subpopulation of cortical pyramidal neurons through the cell’s autonomous Ca^2+^-dependent production of endocannabinoids and K^+^ channel activation^21,22^. Two known endocannabinoids - anandamide and 2-arachidonoylglycerol (2-AG) - show divergent behaviors regarding CB_1_R functionality. Particularly, anandamide, but not 2-AG, inhibits voltage-gated Na^+^ channels located in the intracellular compartments of the cell bodies rather than in the synaptic boutons, whereas presynaptic endocannabinoid activity is predominantly attributed to 2-AG^23^. The endocannabinoid system influences synapse target selection by pyramidal neurons and GABAergic interneurons through CB_1_R internalization from axonal filopodia and chemorepulsion of the growth cones^24,25^. We previously demonstrated that, in cannabinoid type 1 receptor knock-out (CB_1_R^−/−^) mouse embryos, migrating cerebral projection neurons deviated from the vertical orientation, implicating CB_1_R’s role in cellular migration^18^. *In vivo* inhibition of the cannabinoid receptors disrupted migration of immature neurons from the rostral migratory stream in young postnatal mice, as evident by the decreasing length and increasing branching of their leading processes^26^. Cell reorganization during migration, as well as maintenance of the shape of mature cells, are in high degree determined by the cytoskeleton components such as centrosomes, microtubules, actin, myosin II, dynein and others^27–30^. However, the molecular mechanisms of the endocannabinoid system’s action in the cytoarchitecture of the developing brain are still enigmatic.

## Piercing nuclear hernia is a novel ultrastructural pathology of migrating neurons

**Figure 1.**
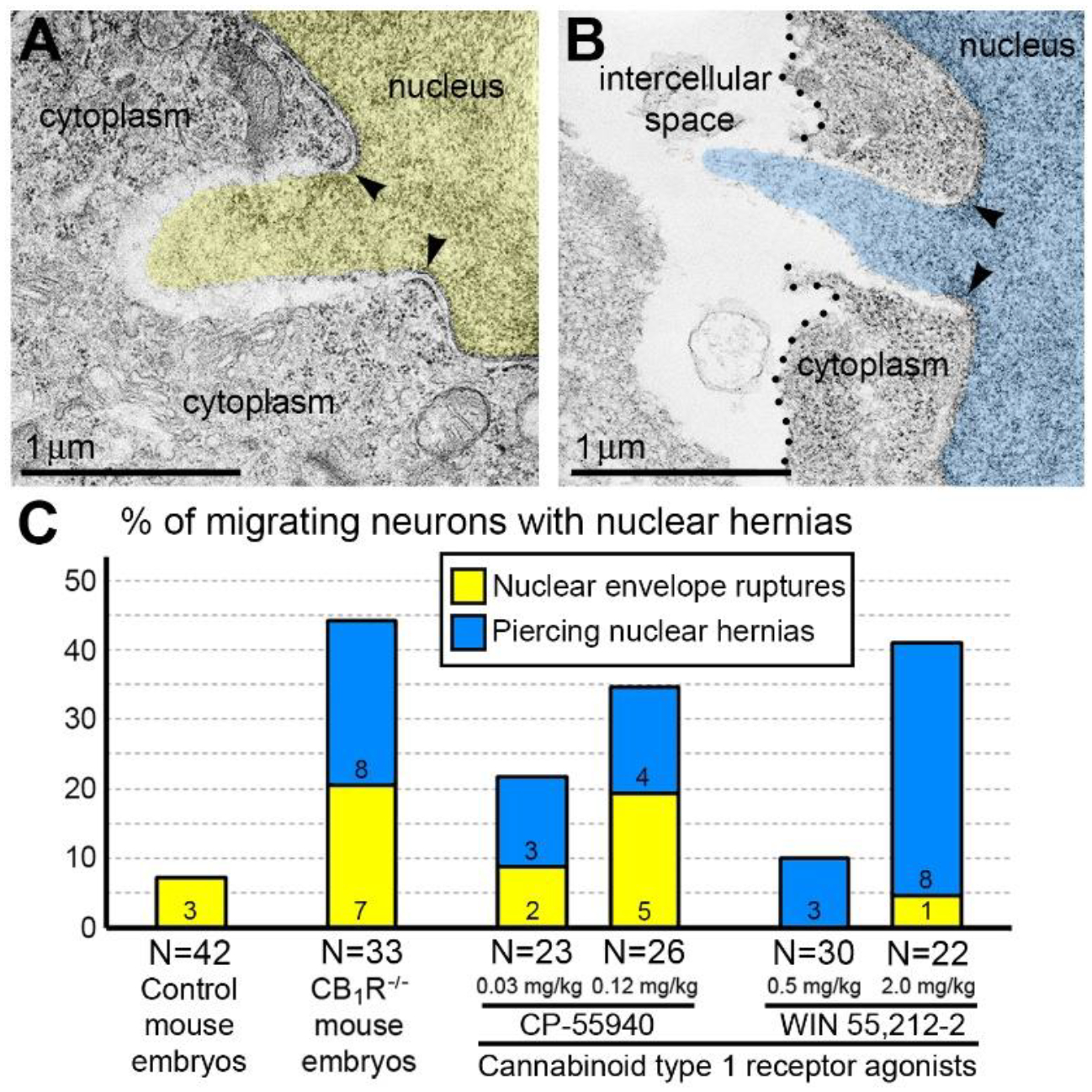
Ultrastructure of herniated chromatin streams and percentage of herniated cells (A) NE rupture in a neuron from the cortical plate of wild type mouse embryo exposed to CB_1_R agonist CP-55940. The chromatin stream expelled from the nucleus (pseudo-colored yellow) is not surrounded by nuclear membranes. (B) Piercing nuclear hernia (PNH) in a neuron from the cortical plate of CB_1_R^−/−^ embryo. The chromatin stream expelled from the nucleus (pseudo-colored blue) penetrates the intercellular space. The damaged plasma membrane is designated with dotted lines in B. Arrowheads in (A) and (B) indicate points of interruption of the nuclear membranes. (C) Percentages of cells with nuclear hernias in the embryo cerebrum of wild type control mice, CB_1_R^−/−^ mice and wild type mice exposed to indicated doses of CB_1_R agonists. Numbers of cells with NE ruptures and PNHs are indicated at the base of each column. Ns refer to the numbers of analyzed cells from corresponding groups. The figure is modified from our article^31^.

Recently, we reported novel ultrastructural pathology in developing neurons related to CB_1_R knock-out and *in utero* application of synthetic cannabinoids^31^. Particularly, we documented numerous local breakups of the NE accompanied by herniation of the nuclear chromatin into the cytoplasm (referred as NE rupture hereafter) in neurons migrating through the mouse embryo cerebrum (Figure 1A). Surprisingly, in a fraction of the cells, streams of herniated chromatin ruptured not only the NE, but also the plasma membrane, exposing cytoplasm and nucleoplasm to the intercellular space (Figure 1B). As it was the first description of simultaneous rupture of the NE and the plasma membrane, we named this cellular pathology ‘piercing nuclear hernia’ (PNH). For detailed characterization of this phenomenon, we performed extensive analyses of electron microscopy imagery of migrating neurons in CB_1_R^−/−^ embryos, wild type control mouse embryos and embryos after *in utero* application of CB_1_R agonists CP-55940 and WIN 55,212–2. Application of two different CB_1_R agonists produced similar chromatin herniation, the extent of which was dose-dependent. Using three-dimensional electron microscopy analysis, we identified 122 nuclear hernias (including 65 NE ruptures and 57 PNHs). In CB_1_R^−/−^ embryos and in wild type embryos shortly exposed to high doses of CB_1_R agonists, about 40% of randomly analyzed migrating projection neurons and interneurons demonstrated NE ruptures or PNHs (Figure 1C). This indicates that deviations from optimal functioning of the endocannabinoid system in either direction – knock-out of CB_1_R or its temporary overstimulation – increase likelihood of altered cellular nucleus and provoke chromatin herniation in a similar way^31^.

**Figure 2.**
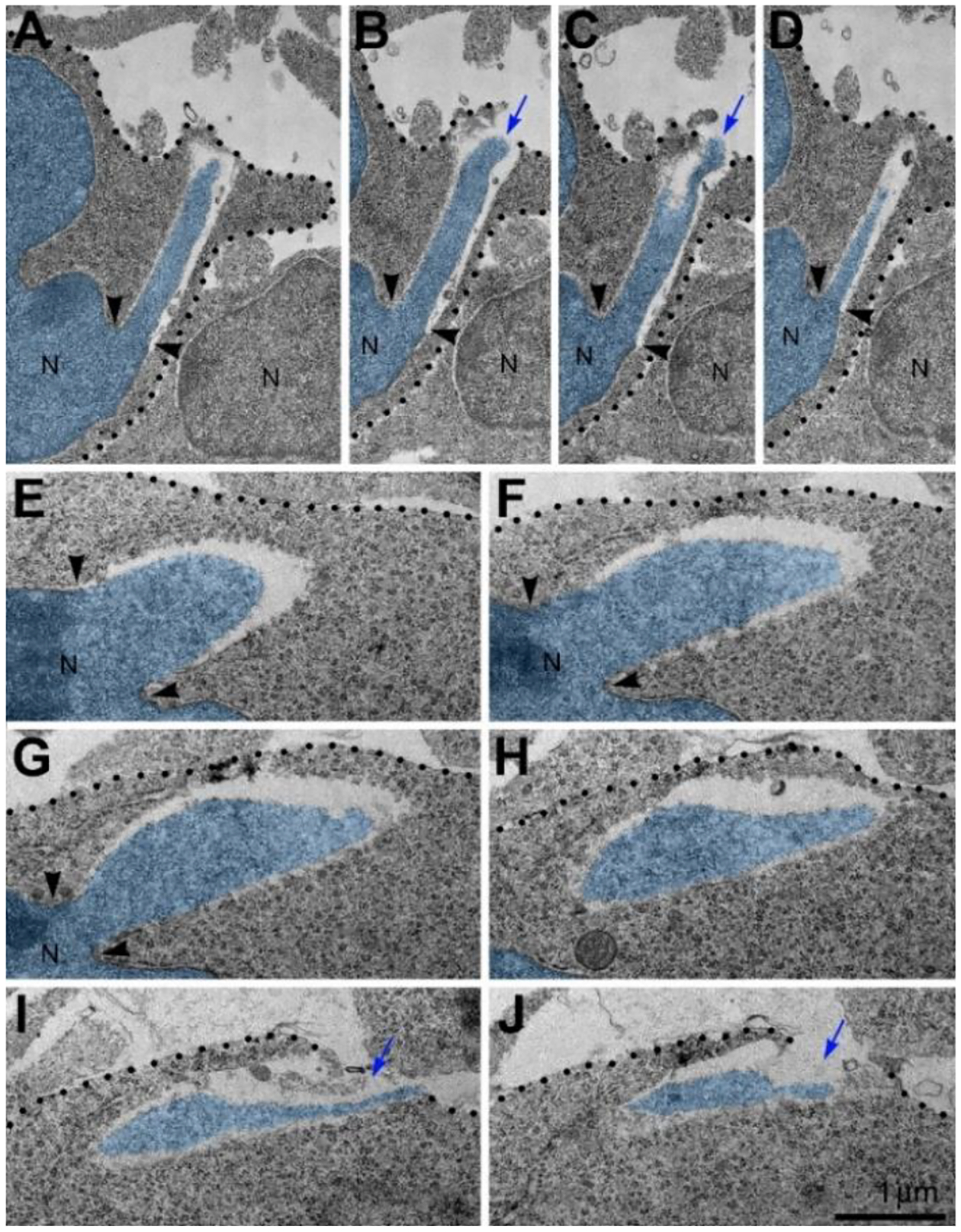
Long length and large volume of herniated chromatin streams are evidence of high intranuclear pressure destroying NE (A-D) Serial micrographs of PNH in a neuron from the cortical plate of CB_1_R^−/−^ embryo show continuum of the chromatin stream expelled from the nucleus (N; pseudo-colored blue) and penetrating the intercellular space. (E-J) Representative set of serial micrographs of PNH in a neuron from the cerebral intermediate zone of an embryo exposed to WIN-55,212–2. Volume of the herniated chromatin stream was estimated in the complete series of 31 sections as 1.63 μm^3^. Blue arrows in (B), (C), (I) and (J) denote the interruption of the plasma membranes, which are designated with dotted lines. Points of NE interruption in (A-G) are indicated with arrowheads. Scale bar in (J) is valid for all. The figure was published in our article^31^.

Morphometric analyses showed that volume of the streams of herniated chromatin varied from 0.02 to 1.63 μm^3^ (Published in^31^). In some analyzed cells, the PNH stream was narrow and reached the length of several microns (Figure 2). Although the molecular mechanism of PNH remains unclear, the large volume and length of the herniated chromatin streams point to abnormally high intranuclear pressure during nuclear translocation - a condition that predisposes cells for NE rupture^32^. Our discovery of a correlation between the disorder of the endocannabinoid system and frequency of NE ruptures and PNHs could be useful for better understanding the effects of cannabinoids in the developing brain (Reviewed in^1,2^). Identification of *in vivo* experimental conditions that increase frequency and power of chromatin herniation may be instrumental for further investigation of the mechanisms of the membrane’s rupture. Here, we review probable cellular mechanisms and developmental consequences of the detected cell pathology as well as the directions of further studies and prospective medicinal application of the obtained knowledge.

## Mechanisms of nuclear envelope rupture and recovery

The nuclear envelope (NE) consists of the inner and outer nuclear membranes and the nuclear lamina - a composite matrix assembled on the inner nuclear membrane. The main function of the NE is to allow molecular exchange between the nucleoplasm and the cytoplasm through specified nuclear pores. Recent works highlighted the dynamic properties of the nuclear membranes and demonstrated that dysregulation of their functions has significant consequences for the cell^33–37^. In addition to biochemical inputs, nuclei of the cells may be exposed to intrinsic and extrinsic mechanical forces transmitted by the cytoskeleton and nucleoskeleton that trigger dynamic changes in nuclear morphology^33,35,36,38^. Ruptures of the NE have been observed in several species *in vitro* and *in vivo* indicating that it is a consequence of the environmental conditions rather than a species-specific phenomenon [Reviewed in^39^]. For example, NE rupture was observed in neurons migrating during brain development^40,41^. NE ruptures were extensively studied in many cancer cell lines during cell migration through tightly constricted areas^34,42–44^. NE ruptures can be induced in non-tumorigenic cell lines by generating a lamin B1 deficiency or depletion of two major tumor suppressors, p53 and retinoblastoma (Rb1)^40,45,46^. Cultivated cells can also be provoked to experience NE rupture by external mechanical force^38,44,47^, human immunodeficiency virus infection^48^, and activation of apoptosis, inflammation, or autophagy^45,49–51^. Emerging data suggests that extensive deformation of the cell and its nucleus during constrained migration transmits substantial physical stress to the NE and may result in its rupture, which in turn leads to chromatin herniation, uncontrolled exchange of nucleocytoplasmic content, DNA damage, and cell death^33,40,42,44,52,53^. Distribution of a diffusible fluorescent marker from a ruptured nucleus through the cytoplasm was observed as early as minutes after NE rupture, indicating the high speed of the reaction^42,43,48,54,55^. The full impact of nucleocytoplasmic mixing is likely to be extensive yet remains poorly understood. Depletion, or mutation of nuclear lamin proteins as well as conditions that impair the connections between the inner nuclear membrane and the chromatin increase the probability of NE ruptures^32,34,35,40,42,43,47,52,56–58^. Thus, NE ruptures may be caused by defects in lamina organization, or increased intranuclear pressure transmitted by actin cytoskeleton and/or nucleoskeleton^32^. Several NE repair mechanisms have been proposed, including attachment of endoplasmic reticulum sheets to the exposed chromatin, spreading of the preserved outer nuclear membrane, plugging with membrane fragments, and resealing by protein complexes [Reviewed in^39,59,60^]. As identified using fluorescent live cell imaging and other methods, BAF (barrier-to-autointegration factor), ECRT-III (the endosomal sorting complexes required for transport-III) and several nuclear membrane proteins such as emerin, LEM-domain containing protein 2 and lamin A/C are involved in the repair of the nuclear envelope^54,61–63^. Cancer and immune cells migrating *in vitro* through constricted microchannels of various sizes reseal their ruptured NE using ESCRT-III and by doing so reduce DNA damage and cell death^42,44^. An individual NE rupture may persist for minutes to hours before repair, however, unrepaired NE ruptures eventually result in the cell’s death^42–44,54,64^.

## Mechanisms of plasma membrane rupture and recovery

Separation of cellular inner content from the environment is one of the fundamental challenges of life due to the variety of mechanical and biochemical stresses that pose constant risks of compromise to the function and viability of the cell. To counteract this threat, eukaryotic cells have developed efficient repair mechanisms, which are heavily conserved across species and seem to have co-evolved with the emergence of vital properties of the plasma membrane^65–68^. Membrane repair mechanisms include distinct reactions depending on the size of the rupture [Reviewed in^66,68,69^]. Namely, tiny membrane injuries (less than a nanometer) may be repaired spontaneously by reciprocal attraction of the hydrophobic domains of lipids. Injuries larger than a few nanometers in diameter, such as those caused by bacterial pore-forming toxins, require the help of an active membrane repair mechanism that includes endosomal uptake or exosomal secretion of the bacteria-lined pores. Repair of very large disruptions of the plasma membrane (hundreds to thousands of nanometers in diameter) requires complicated vesicle-vesicle and vesicle-plasma membrane fusion mechanisms. The initial repair steps include plugging the membrane rupture with conglomerates of vesicles or membrane whorls that crudely ‘patch’ the rupture, restricting the leak of cytoplasm and providing time for reconstruction of intact plasma membrane^66,68–70^. A major trigger for the signaling cascade that precedes membrane resealing is the influx of Ca^2+^ from the intercellular space through the membrane disruption. Subsequent vesicle deposition and fusion involves complex Ca^2+^-dependent pathways acting through the cAMP signaling cascade, cytoskeleton reconstruction, SNAP receptors, the phospholipase enzymes, calpain proteases and others. The membrane patch serving a temporary barrier is subsequently remodeled or removed via exocytic or endocytic machinery^66,68,69^. Alternatively, if the damaged plasma membrane is not repaired within minutes, activation of apoptotic or necrotic pathways mediated through Ca^2+^ influx often results in cell death^69,71^. Repair mechanisms of large membrane ruptures are effective for certain cell types, e.g., muscular cells, oocytes, epithelial cells, and invasive cancer cells^69,70^. While neurons may effectively restore transected axons, recovery of neuronal cell bodies following severe ruptures of plasma membranes has not been demonstrated^65^.

## Probable death or survival of neurons with piercing nuclear hernia

PNH detected in migrating neurons catastrophically breaks isolation of the inner content of the cell from the environment^31^. Cells exposed to extreme physical, chemical, or mechanical stimuli may immediately lose their structural integrity and die in an uncontrollable manner termed ‘accidental cell death’, which is opposite to mechanisms of programed cell death such as apoptosis, autophagy, or necrosis-type cell death^65,72–75^. Cases of accidental cell death are virtually undetectable using biochemical or immunochemical methods such as TUNEL staining, caspase immunolabeling, or labeling of autophagy markers. Electron microscopy visualizes organelles regardless of their functional conditions and can detect dead cells or cellular remnants^75^.

**Figure 3.**
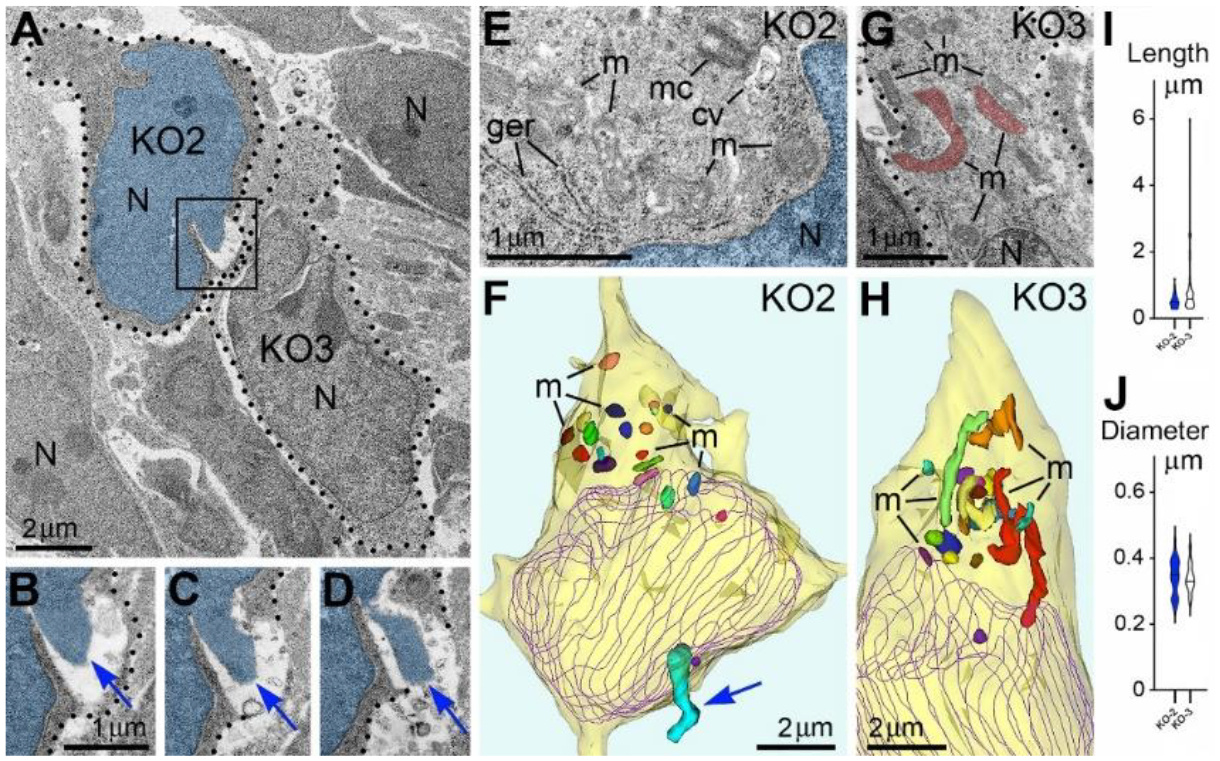
Adjacent herniated and non-herniated neurons show distinct ultrastructural characteristics of mitochondria (A) Two neurons from the intermediate zone of CB_1_R^−/−^ embryo indicated as cells number KO2 and KO3 (dotted lines designate their plasma membranes). Cell KO2 exhibits PNH whereas KO3 shows an intact nucleus. (B-D) Serial images of PNH from the framed area in (A). The chromatin stream (blue arrows) herniated from the nucleus (pseudo-colored blue) penetrates the intercellular space. Scale bar in (B) is valid for (C) and (D). (E-H) High power images (E) and (G) and 3D reconstructions (F) and (H) of the cells KO2 and KO3. 20 randomly selected mitochondria from each cell are shown in different colors in the 3D reconstructions. Cell KO2 exposing PNH (blue arrow) contains mostly short or spherical mitochondria, whereas several mitochondria from cell KO3 are long. Nuclei profiles are traced violet. Abbreviations: cv, cilial vesicle; ger, granular endoplasmic reticulum; m, mitochondria, mc, mother centriole; N, nucleus. (I, J) Estimation plots of lengths and diameters for mitochondria from cells KO2 (blue) and KO3 (white). The figure was published in our article^31^.

**Figure 4.**
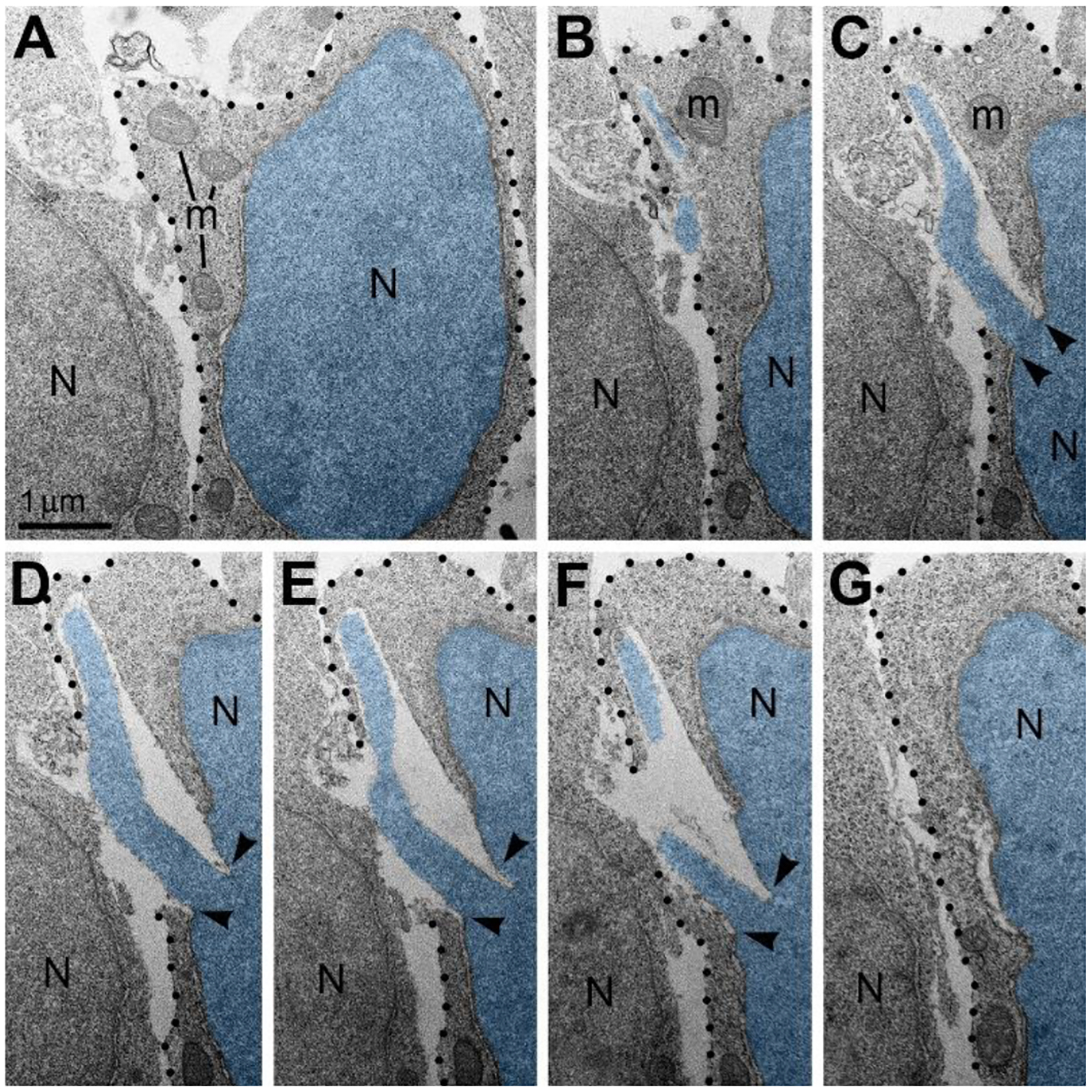
An example of plasma membrane rupture blocked by adjacent cell (A-G) Serial electron micrographs depict a neuron with PNH from the cortical plate of wild type mouse embryo exposed to CB_1_R agonist WIN 55,212–2. The nucleus (N) and PNH stream are pseudo-colored blue. Points of interruption of NE are indicated with arrowheads. The plasma membrane is designated with dotted lines. Although the nuclear hernia produces a large rupture of the plasma membrane, the cellular cytoplasm does not show evidence of degradation and mitochondria (m) do not show evidence of swelling. Scale bar in (A) is valid for all. The figure was published in our article^31^.

Given the high frequency of cells with NE rupture and PNH in certain experimental conditions (Figure 1C) one might expect to detect many dead cells. Surprisingly, we did not observe a considerable number of cellular remnants or cells with ultrastructural features of necrotic, apoptotic or autophagy type degradation in the embryos containing numerous cells with herniated nuclei. We also did not identify massive swelling of the mitochondrial matrix that was recognized to be irreversible and foreshadowing cell death through a necrotic mechanism^76,77^. Instead, a fraction of the cells showed reduction of mitochondrial length – evidence of mitochondrial fission. It is known that morpho-functional conditions of mitochondria correlate with general cellular functionality, while predominance of mitochondrial fission over fusion may serve as evidence of disordered cellular energetics or other moderate malfunctions^76–82^. We often observed dramatic heterogeneity of ultrastructure between certain cells from the same sample. For example, cells with PNH demonstrated short mitochondria while adjacent non-herniated cells showed normally sized mitochondria (Figure 3). At the same time, other cells with PNH showed normal ultrastructure of mitochondria and other organelles^31^. Different time courses of NE rupture (likely occurring in the span of seconds^42,43,48,54,55^) and mitochondrial reaction (delay in the span of hours^76^) may partially explain the divergence between emergence of PNH and reaction of organelles in the embryos temporarily exposed to CB_1_R agonists. Nevertheless, the absence of a considerable number of dead cells in CB_1_R^−/−^ embryos, which constantly experience disorder of endocannabinoid signaling, raises a supposition that PNH may be repaired rather than always be fatal for the cell. This observation aligns with the fact that CB_1_R^−/−^ mice generally do not exhibit obvious behavioral phenotypes^83^. In accordance, partial recovery of cells after NE rupture has been documented^39,59^. Surviving neurons were reported after transection of the axons, but not after damage of the cell bodies^65^. We considered morphological features that might promote recovery of migrating neurons with PNH. Namely, for a fraction of the cells with PNH, the area of the plasma membrane rupture was closely surrounded by adjacent cell bodies or processes, which may contribute to decreasing the flow of cytoplasm and blocking the leak (Figure 4). Although the effectiveness of such blocking is difficult to estimate in direct experiments, reduction of the cytoplasm leak because of ‘patching’ by adjacent cells, together with a possible delay of the organelles’ reaction, may explain dissimilarity of the ultrastructure in several cells with PNH. Thus, although death of cells with ruptured NE and plasma membrane is probable, PNH in mouse embryo brains may be non-fatal for a fraction of the affected cells. This suggests the possibility of recovery for catastrophically damaged neuronal cell bodies, which has not been demonstrated previously. Deeper study of the newly detected role of the endocannabinoid system in rupture and recovery of cell membranes could uncover presently unknown molecular mechanisms that prove to be instrumental for increasing regenerative capacity of neuronal cell bodies^65,66,68,70,71,84^.

## Nuclear envelope ruptures and piercing nuclear hernias suggest role of the endocannabinoid system on cytoskeleton functionality in cell bodies

High percentages of cells with nuclear hernias in the embryo cerebrum of CB_1_R^−/−^ mice and wild type mice exposed to CB_1_R agonists CP-55940 and WIN 55,212–2 (Figure 1C) indicate involvement of the endocannabinoid system in the mechanics of NE rupture^31^. Judging from the large volume and the length of herniated chromatin streams, the NE ruptures and PNHs in the nuclei translocating through tightly packed tissue such as the developing mammalian cerebrum are likely consequences of increased intranuclear pressure and suboptimal function of the cytoskeleton^32,52^. In accord, chronic treatment of adult rats with WIN 55,212–2 increased expression of neurofilaments Nf-160 and Nf-200, and microtubule-associated protein-2 (MAP-2). Meanwhile, CB_1_R^−/−^ mice demonstrated a lower expression of the neurofilaments and MAP-2, along with reduced dendritic arborization in the hippocampus^85–87^. Such deviated expression of cytoskeletal components after stimulation or knock-out of CB_1_R establishes evidence of cannabinoid signaling participating in neuronal cytoskeleton consolidation. Direct link of endocannabinoid system, and particularly CB_1_R, with the function of the cytoskeleton was demonstrated in the context of axonal pathfinding. This mechanism involves CB_1_R internalization from filopodia and chemorepulsion of the axonal growth cones by activating RhoA GTPase (small guanosine triphosphatase) and Rho-associated kinase (ROCK)^24^. RhoA GTPases are known for their role as molecular switches transducing stimuli to the actin cytoskeleton^88,89^. CB_1_R-activation rapidly and reversibly contracts the neuronal actomyosin cytoskeleton through coupling to G_12_/G_13_ proteins that produce Rho- and ROCK-mediated non-muscle Myosin II (NM II) activation in the axons of cultured neurons^90^. Early neurula stage chicken embryos and early tailbud stage frog embryos exposed to CB_1_R agonist ACEA (arachidonyl-2′-chloroethylamide) or CB_1_R inverse agonist AM251 demonstrated disordered migration of neural crest cells. The effects of deviated CB_1_R signaling in over- and under-activity were mimicked by exposing the embryos to NM II inhibitor Blebbistatin, which indicated that CB_1_R regulated migration of neural cells in chicken and frog embryos through cytoskeleton functionality^91^. Thus, the unknown role of CB_1_R in the developing brain of many different species may include maintaining optimal function of the actomyosin cytoskeleton, which is crucial for translocation of neuronal cell bodies.

## Emergences of nuclear envelope ruptures and piercing nuclear hernias suggest role of cannabinoid type 1 receptor in neuron migration

Early expression of CB_1_R indicates its probable involvement in prenatal and postnatal development of the mammalian brain^17,19^. Numerous studies indicate that prenatal inhibition or overstimulation of the endocannabinoid system may provoke long-term consequences in the offspring, for example, altered breathing, disturbed suckling, and memory deficit linked to disorder of glutamate-ergic neurons^2,12,92–95^, but many aspects of the underlying mechanisms remain enigmatic. Our data indicates that disorder of the endocannabinoid system may provoke NE ruptures and PNHs in migrating neurons^31^. Although we did not document dead or severely damaged cells, our study does not assume a complete recovery and absence of a long-term effect on the cells experiencing NE ruptures or PNHs. More likely, temporary damage of NE and plasma membrane provokes a delay or disorientation of the cell migration. A body of published evidence supports the involvement of cannabinoid signaling in migration of immature neurons. Particularly, inhibition of cannabinoid receptors or DAGL (diacylglycerol lipase, the enzyme synthetizing the endocannabinoid 2-AG) decreases migration of cultivated neural stem cells and neuroblasts from the rostral migratory stream explant culture. Consequently, activation of cannabinoid receptors or preventing breakdown of 2-AG increases migration^26^. Early post-natal CB_1_R^−/−^ mice display higher numbers of projection neurons in deeper cortical layers and lower numbers in the superficial layers identifying altered neuronal migration^96^. Similar mis-location of neurons was detected in fatty-acid amide hydrolase (enzyme degrading endocannabinoid anandamide) knock-out mice (FAAH^−/−^) indicating that supposed overstimulation of CB_1_R through increased amount of anandamide may also disorder cellular migration^96^. Acute knockdown of CB_1_R by *in utero* electroporation of siRNA in mouse embryos delayed colonization of cortical plate by projection neurons; instead, many cells were stacked in the intermediate and subventricular zones^97^. Thus, delay in radial migration of neurons in the absence or altered functionality of CB_1_R may be the reason for previously identified moderate disorder of cerebral cytoarchitecture and brain dysfunction in CB_1_R^−/−^ mice, or cognitive deviations in animals and humans exposed to cannabis during development^1,2,12,83,92–95^.

Our quantitative immunohistochemical analyses show the probability (rather than unequivocal proof) of a link between chromatin herniation and the amount of CB_1_R in the cell body^31^. This leaves the point unsolved, whether cannabinoid signaling disorder upregulates NE ruptures and PNHs directly in the CB_1_R-expressing cells, or the action is systematic, for example, through vasodilation and hypotensive effects in the entire fetus, placenta or maternal organism^1,2,9,10^. Accordingly, mouse embryos from pregnant dams exposed to cannabinoid agonist CP-55,940 combined with alcohol vapors demonstrated reduced internal carotid artery blood flow and significant subsequent perinatal mortality; the survived young adult offspring showed induced deficit in neurobehavioral motor outcomes^10,98^. Our finding opens an opportunity for further investigation if temporary disorder of the endocannabinoid system, for example, in cases of recreational cannabis use by pregnant women, may provoke accidental death or malfunction in a fraction of migrating neurons.

## Prospective study of cannabinoid system and piercing nuclear hernia in aspects of medicinal application

Therapeutic benefits of cannabis as an analgesic and antiemetic agent are well-known and applied for centuries^3,99^. Cannabinoid-based anti-epileptic and anti-spasticity medications such as Epidiolex and Nabiximols (Sativex) have demonstrated efficacy in randomized controlled trials^100,101^. Inhibition of MAGL (monoacylglycerol lipase – enzyme degrading endocannabinoid 2-AG) has been proposed as a potential therapeutic approach for treatment of diverse neurological and neurodegenerative diseases, such as multiple sclerosis, Alzheimer’s disease, Parkinson’s disease, amyotrophic lateral sclerosis, and traumatic brain injury^102–108^. Our finding of involvement of the endocannabinoid system in the membrane’s rupture and probably in the recovery of the disordered cells^31^ may help to reveal potential neuroprotective action of cannabinoids.

Two cannabinoid drugs (dronabinol and nabilone) are approved by the U.S. Food and Drug Administration for the prevention or treatment of cancer-related side effects, such as nausea and vomiting. Numerous *in vitro* and *in vivo* experiments on cancer models show that cannabinoids can kill cancer cells, making cannabis-based medicine a promising option to effectively modulate growth of certain tumor types. The observed effects of the cannabinoids’ application include inhibiting tumor growth and metastasis, reducing cell viability by promoting apoptosis, and inhibiting angiogenesis; however, the specific molecular mechanisms at play were not identified in detail. Accordingly, suspected anticancer benefits of cannabinoids have yet to be confirmed in clinical trials^109–118^. Our findings suggest a novel mechanism of cannabinoid action on the nuclei of migrating cells and, after additional study, may be instrumental for inducing breaks of the plasma membrane in metastatic tumor cells. Although NE rupture resulting in genomic instability might promote cancer progression, unstable NE also represents a particular weakness of metastatic cancer cells. Accordingly, NE ruptures detected in many cancer cell lines during cell migration through tightly constricted areas often result in cell death^42–45^. Conditions that have potential to increase the probability of NE rupture and death of metastatic cells are promising as cancer treatments^34,42^. We suggest performing extensive investigation of conditions of PNH generation for the purpose of targeting metastatic cancer cells. The conditions increasing frequency and power of NE ruptures may include manipulations of the endocannabinoid system and probably other molecular mechanisms. This opens an opportunity for a new research direction that may result in the development of therapeutic applications of cannabinoids for targeting cancer cells.

## Conclusions

Neurons migrating through the developing cerebral cortex show nuclear envelope ruptures and herniations of the chromatin in cytoplasm. Powerful streams of herniated chromatin may also pierce plasma membranes and expose nucleoplasm and cytoplasm to the intercellular space representing a novel form of ultrastructural cell pathology. Knock-out or over stimulation of the cannabinoid type 1 receptor increases probability and power of the nuclear envelope rupture and chromatin herniation. As previously demonstrated, nuclear envelope ruptures result from disfunction of the cytoskeleton. Thus, deviation of endocannabinoid signaling from optimal functionality in either direction likely provokes disfunction of the cytoskeleton in migrating neurons. Catastrophic damage of the main cellular membranes normally results in accidental cell death. In perspective, this newly discovered pathology of migrating cells and technique of its upregulation may be applied for inducing breaks of the plasma membrane and death of metastatic tumor cells. On the other hand, observed evidence of recovery of neuronal cell bodies, after further study, may become instrumental for increasing regenerative capacity of damaged neurons.
